# Structure-Guided Glycosylation of Hemagglutinin Enhances Stability and Modulates Immunogenicity of Influenza Vaccines

**DOI:** 10.3390/vaccines14050443

**Published:** 2026-05-15

**Authors:** Zheng Zhang, Zhiying Xiao, Xu Zhang, Qian Ye, Xin Zhang, Wen-Song Tan

**Affiliations:** 1 State Key Laboratory of Bioreactor Engineering, East China University of Science and Technology, Shanghai 200237, China; y30230576@mail.ecust.edu.cn (Z.Z.); y12243109@mail.ecust.edu.cn (Z.X.); zhangxu@ecust.edu.cn (X.Z.); qy@mail.ecust.edu.cn (Q.Y.); y16240064@mail.ecust.edu.cn (X.Z.); 2 Shanghai Collaborative Innovation Center for Biomanufacturing Technology (SCIBT), Shanghai 200237, China; 3 Department of Virology & Vaccine, Shanghai Institute of Biological Products, Shanghai 200052, China; 4 Shanghai BioEngine Sci-Tech Co., Ltd., Shanghai 201203, China

**Keywords:** hemagglutinin, glycosylation, H1N1, influenza virus, vaccines

## Abstract

Background: Antigenic drift limits the protective efficacy of influenza vaccine. Glycosylation of hemagglutinin (HA) represents a promising immunofocusing strategy that enhances neutralizing antibody responses by masking immunodominant non-neutralizing epitopes. Methods: B-cell epitopes of influenza viruses were retrieved from the Immune Epitope Database and were mapped onto the HA structure of A/Puerto Rico/8/1934 (H1N1). Structure-guided analysis identified residues 136 and 137 as candidate sites for N-linked glycosylation (NLG). Single-site mutants (136NLG and 137NLG) were generated using reverse genetics and evaluated for stability, receptor binding, viral replication, and immunogenicity in a murine model with inactivated whole-virus vaccines. Results: Both mutants exhibited increased thermostability at 42 °C. Glycosylation reduced the HA–sialic acid affinity, resulting in decreased viral adsorption and internalization efficiency in MDCK cells, and delayed viral replication at low multiplicity of infection (MOI). In vivo, all vaccine groups provided complete protection against lethal challenge; notably, the 136NLG group exhibited reduced weight loss, indicating improved protective efficacy compared with wild-type (WT). Conclusions: Targeted glycosylation at residue 136 in the HA head domain effectively enhances the viral stability and elicits a 1.78-fold increase in hemagglutination inhibition titer (GMT) relative to the WT, thereby improving vaccine performance. These findings establish a rational and structure-based design strategy for developing more stable and effective influenza vaccines.

## 1. Introduction

Influenza A virus (IAV) has been a global pandemic threat to both human and animals for over a century, causing at least four global pandemics [1,2]. Antigenic drift substantially reduces vaccine efficacy, necessitating strategies that enhance immunogenicity and direct protective antibody responses. Conventional strategies, such as dose escalation and adjuvant incorporation, provide limited improvement [3]. More recently, immunofocusing has emerged as a strategy to preferentially direct humoral responses toward protective epitopes [4].

Hemagglutinin (HA), the most abundant surface glycoprotein of influenza virus, mediates viral attachment and membrane fusion and is the primary target of neutralizing antibodies. Approximately 300–350 HA trimers are present on each virion [5]. Neutralizing antibodies primarily inhibit viral entry by blocking HA-receptor interactions, making HA the principal antigen in most influenza.

N-linked glycosylation, a common post-translational modification in which glycans are attached to asparagine residues within the Asn-X-Ser/Thr-Y motif, where X/Y may represent any amino acid except proline, plays a critical role in viral immune evasion [6]. Influenza viruses frequently acquire or reposition glycosylation sites to escape host immunity [7]. Due to their steric bulk and conformational flexibility, glycans can shield immunodominant but non-neutralizing epitopes, thereby redirecting immune responses. Previous studies have demonstrated that hyperglycosylation can enhance neutralizing breadth and modulate antigenicity, supporting glycan-mediated immunofocusing as a viable vaccine design strategy [8,9,10].

Despite these advances, current glycosylation sites selection largely relies on evolutionary analyses or data-intensive computational predictions, which may not optimally enhance immunogenicity. Therefore, a rational, structure-guided approach integrating epitope distribution and protein function is needed.

In this study, we developed an epitope-informed, structure-guided glycosylation strategy targeting the HA head domain. By introducing a single N-linked glycosylation site based on epitope mapping and structural analysis, recombinant viruses were generated using reverse genetics. Glycosylation enhanced thermal and pH stability, reduced HA–sialic acid binding affinity, and consequently decreased viral entry efficiency and replication in MDCK cells. In a murine model, inactivated vaccines derived from the glycosylated variant elicited stronger humoral responses, with increased hemagglutination inhibition and microneutralization antibody titers compared with the wild-type strain.

In summary, this study establishes a rational, structure-based design strategy that enhances antigen stability and focuses immune responses toward protective epitopes within the receptor-binding site. Validation in the PR8 model demonstrates that the 136NLG mutant improves immunogenicity and protective efficacy, providing a promising framework for next-generation influenza vaccine design that may have the potential to mitigate original antigenic sin.

## 2. Materials and Methods

### 2.1. Cells Culture and Plasmids

Adherent MDCK (ATCC, CCL-34) and HEK-293T (ATCC, CRL-3216) cells were cultured in T-flasks and maintained at 37 °C in a humidified incubator with 5% CO_2_. The growth medium was DMEM (BioEngine Sci-Tech, CN) containing 10% (*v*/*v*) fetal bovine serum (Biosun, Shanghai, China).

As shown in Figure S1, eight plasmids (CH-PB2, CH-PB1, CH-PA, CH-M, CH-HA, CH-NA, CH-NP, and CH-NS) were prepared for the PR8 strain—a wild-type H1N1 IAV (A/Puerto Rico/8/1934). Each of these constructs contains one segment of the IAV genome, with both a Polymerase I promoter–terminator pair and a Polymerase II (CAG) promoter–terminator pair flanking the segment.

Glycosylation was introduced at a specific site via a single-point mutation in the CH-HA plasmid, with the mutation confirmed by sequencing. Precise mutations are shown in Figure S2.

### 2.2. Virus Rescue and Analysis of Genetic Stability

To evaluate the ability of the tested cells to generate infectious viral particles, plasmids encoding the missing viral components were co-transfected into the cells. All transfections were carried out using PEI MAX MW40000 (Polysciences, Warrington, PA, USA) in DMEM, following the supplier’s protocol. Twelve hours later—allowing sufficient time for plasmid entry and expression—the medium was replaced with fresh DMEM to wash away residual plasmids. At 48–72 h post-transfection (hpt), the culture supernatant was harvested, and viral titers were determined by TCID_50_ assay. For samples showing low titers, viral production was assessed qualitatively by overlaying MDCK cells onto the culture wells at 48–72 hpt in the presence of TPCK trypsin (3 μg/mL), prior to titration. As a positive control, the PR8 virus was rescued by transfecting eight plasmids into HEK-293T cells. The presence of IAV particles released into the supernatant was rapidly confirmed using a hemagglutination (HA) assay [11,12].

The genetic stability of the rescued viruses was assessed over 10 passages in MDCK cells. Infected cultures were incubated at 37 °C for 72 h, after which viral stocks were harvested and their HA genes sequenced [13].

### 2.3. TCID_50_ and HA Assay

The TCID_50_ assay was conducted as described previously. Instead of directly observing cytopathic effects (CPEs), HA assays were employed to identify virus-positive wells. Infectious virion counts per mL were calculated using the Reed–Muench method. The TCID_50_ titer of the seed virus used for infection was determined on adherent MDCK cells.

HA Assay was measured in round-bottomed 96-well microtiter plates (Beyotime Sci-Tech, CN). Briefly, each sample was subjected to √2 -fold serial dilutions. Equal volume (100 μL) of purified chicken erythrocytes was added to each well and plates were incubated at room temperature (RT) for at least 2 h up to overnight. After scanning the plates at 700 nm with a plate photometer, extinction data were processed in Origin software. Extinction was plotted as a function of the negative log dilution, and a Boltzmann sigmoid was fitted to each curve by non-linear least squares. The titration endpoint was taken as the dilution at the inflection point, which is one of the fitted parameters. The inverse of the dilution was defined as the specific HA activity with units 1 HAU/100 μL [14].

The Rapid HA assay was carried out using V-bottomed 96-well microtiter plates. Briefly, two-fold serial dilutions of the virus in PBS were prepared, and mixed each dilution with 25 μL of purified chicken erythrocytes. The mixture was incubated for 30 min at RT. HA titers were determined as Log_2_HA units per test volume (Log_2_HAU/25 μL) [15].

### 2.4. Sequence Alignment and Protein Visualisation

The HA gene sequences of H1N1 IAVs were downloaded from the GISAID (http://platform.gisaid.org, accessed on 30 April 2025) database (data as of 30 April 2025). High-similarity sequences were removed using BioAider software 1.727 (99%) [16], and the resulting sequences were aligned using the MAFFT tool (*n* = 2856) [17]. The analysis of glycosylation sites was conducted utilizing the N-GlycoSite tool from the HIV (https://www.hiv.lanl.gov/content/index, accessed on 30 May 2025) database [18]. Glycosylation sites in the PR8 strain sequence were predicted using NetNGlyc-1.0 [19].

Downloaded positive results for epitopes identified in influenza viruses (NCBI Taxon: 11320) from the IEDB database [20], utilizing Python 3.9.7, the sequence of the HA protein from the PR8 strain was aligned against the epitope. Each amino acid was assigned a score and normalized, and then written into the B-factor column of the PDB file (1RU7). The HA1 subunit in the PDB file was visualized using coloring in PyMOL 3.0.4. This analysis is limited to linear epitopes and does not account for conformational epitopes.

Algorithm schematics and raw data are provided in the Supplementary file. The computational case scripts are available on GitHub. (https://github.com/zzone7/IAV-PR8.git, accessed on 1 May 2026).

### 2.5. GlycoSHIELD Simulation and Stability Testing

Analysis of the glycosylation sites of the H1N1 strain gave us a map of the amino acid sites of glycan modification, and in order to assess the effect of glycan masking with the addition of glycan chains, simulations were carried out using an online tool using the high-mannose glycoform (Man5Fuc1) as the simulated grafted glycan chain, a glycoform that is common in influenza viruses produced by MDCK [21].

pH stability assay: Each buffer—100 mM acetate (pH 4.0 or 5.0), 100 mM phosphate (pH 6.0), or 100 mM neutral phosphate (pH 7.0)—was mixed with an equal volume of virus at 128 HAU/25 μL. After a 10 min incubation at 37 °C, the mixture was subjected to a Rapid HA assay to determine viral titers [22].

Thermostability assay: Samples containing 128 HAU/25 μL of virus were placed at either 37 °C or 42 °C. Incubation lasted 4, 8, 24, 48, or 72 h, after which the heated samples were chilled to 4 °C, and viral titers were determined by the Rapid HA assay [23].

### 2.6. Western Blotting Assay

The occupancy of glycans within the predicted N-linked glycosylation sites (NGSs) on HA was determined by co-incubating the HA protein—engineered with a defined glycosylation pattern—with PNGase F (Beyotime Sci-Tech, Shanghai, China) under denaturing conditions, as per the manufacturer’s guidelines, to achieve complete glycan removal. Briefly, The virus infected MDCK with MOI = 1, and after 18 h, the cells were lysed using Western/IP Cell Lysate (Shanghai Life-iLab Biotech Co., Ltd., Shanghai, China) for supernatant, and a portion of the samples was taken and treated with PNGase F. Samples were subjected to a Western blot analysis, probed with Influenza A H1N1 (A/Puerto Rico/8/1934) HA Antibody, Rabbit pAb (Sino Biological, Beijing, China) against the HRP-labeled Goat Anti-Rabbit IgG(H+L) (Beyotime Sci-Tech, Shanghai, China). Band shift patterns served to confirm whether glycosylation was present or absent at differing N-glycosylation sites (NGSs) among the mutants [24].

### 2.7. Virus Adsorption and Internalization Efficiency

Briefly, the adsorption efficiency was characterized by the reduction in the number of viral particles in the supernatant before and after synchronous infection with high MOI(MOI = 10). The number of virus particles was calculated according to Equation (1) [25].

To support the experimental results, Viruses were incubated with 1% chicken erythrocytes at 4 °C for 2 h. The absence of hemagglutination in the supernatant was detected to ensure that all viruses were adsorbed to chicken erythrocytes, and the samples were then transferred to 33 °C to allow virus release. After 30 min, the supernatants were centrifuged to detect HA, and changes in the adsorption efficiency (affinity for sialic acid) of the viral variants were characterized by differences in HA titers [26].

(1) n(virions)=n(chicken erythrocytes)×HA

For internalization efficiency, MDCK cells were seeded in 96-well plates and cultured overnight to adhere. Prior to viral exposure, the plates were pre-cooled for 1 h at 4 °C. Ice-cold complete medium containing IAV virions was then added, and the cells were kept at 4 °C for 2 h. The cells were then washed 3 times in ice-cold PBS to remove unbound virions and either fixed directly or first incubated at 37 °C in complete medium to allow virion endocytosis. Fixation was performed by adding ice-cold 4% formaldehyde in PBS and leaving the plates at room temperature (RT) for 1 h. The fixation reaction was terminated by a 15 min RT incubation with 0.1 M glycine in PBS, followed by three PBS rinses. Following permeabilization (0.1% Triton X-100 in PBS, 30 min, RT), cells were blocked overnight at 4 °C with 2% BSA-0.1% Tween 20 in PBS. A 3 h RT incubation with HA-antibody (1:1000 in blocking buffer) ensued, after which the cells were washed three times in PBS containing 0.1% Tween 20. A second 3 h RT incubation with HRP-IgG (1:2000 in blocking buffer) was then performed, concluding with six washes in PBS-0.1% Tween 20. Color development using TMB Chromogen Solution (Beyotime Sci-Tech, Shanghai, China), measuring light extinction at 370 nm. All absorbance values were normalized to the values measured for virus bound at 4 °C [27].

### 2.8. Viral Growth Kinetics

Briefly, MDCK cells in 6-well plates were infected with each virus at different MOI. Cell supernatants were collected every 24 h for 72 or 96 h post-infection to determine the HA titer at each time point.

### 2.9. Immunogenicity of the Recombinant Viruses

A total of 36 female 6-8 week-old BALB/c mice were randomly assigned into 4 different groups: PBS, WT, 136NLG, 137NLG (*n* = 9 per group), and received a single immunization via unilateral quadriceps injection of 50 μL of inactivated whole-virus vaccine (0.135 μg HA/dose). The virus was inactivated by adding 0.05% β-propiolactone (*v*/*v*) and incubating at 4 °C for 24 h. Two weeks post-immunization, six immunized mice per group underwent blood collection for antiserum preparation, while the remaining three underwent challenge experiments with 10 LD_50_ of PR8 virus. The mice were monitored daily for 7 days and body weight and survival were recorded. The animals that had lost more than 20% of their initial body weight were euthanized and recorded as dead.

### 2.10. Haemagglutinin-Inhibition (HI) Assay and Microneutralization (MN) Assay

After pre-treatment with receptor-destroying enzyme (RDE, Denka Seiken, Niigata, Japan). The mouse serum samples were subjected to an HI assay performed with 4 hemagglutination units (HAU) of H1N1 virus and a 1% (*v*/*v*) suspension of chicken erythrocytes to detect the inhibition of hemagglutination by antibodies in the serum.

The MN assay was performed as previously described [28]. Sera were subjected to twofold serial dilution in PBS from an initial 1:10 dilution. Each diluted sample was then combined with 100 TCID_50_ of PR8 virus and kept at 37 °C for 1 h. After that, MDCK cells received the serum–virus mixture and were kept for 1 h of incubation. Following incubation, the serum–virus mixtures were removed. The cells were supplied with serum-free DMEM (2 µg/mL TPCK-trypsin) and subsequently kept at 37 °C with 5% CO_2_. To determine the MN titer, the supernatant collected at 72 h post-incubation was mixed 1:1 with a 1% (*v*/*v*) suspension of chicken erythrocytes, allowing detection of viral hemagglutination. The titer corresponded to the greatest dilution of serum at which no hemagglutination occurred [29].

### 2.11. Statistical Analysis

All values in the figures are shown as mean ± standard deviations (SD). The data were analyzed using one-way ANOVA in GraphPad Prism 10.1.2 Software. When calculating the fold change in antibody titers, the data are first subjected to logarithmic transformation, followed by Tukey’s test for multiple comparisons to compute the corresponding p-value. Asterisks in the figures denote statistically significant differences (*, *p* < 0.05; **, *p* < 0.01; ***, *p* < 0.001; ****, *p* < 0.0001).

Unless otherwise noted, all experiments consisted of at least three independent biological replicates.

### 2.12. Ethics Statement

Approval for the animal experimental protocol (No.2025007) was obtained from both the Animal Management Committee and the Animal Ethics and Welfare Protection Group of the Shanghai Institute of Biological Products. All procedures involving animals were carried out in compliance with the animal ethics guidelines established by the National Health and Medical Research Council of China.

## 3. Results

### 3.1. Selection of HA1 for Glycosylation Modifications

**Figure 1:**
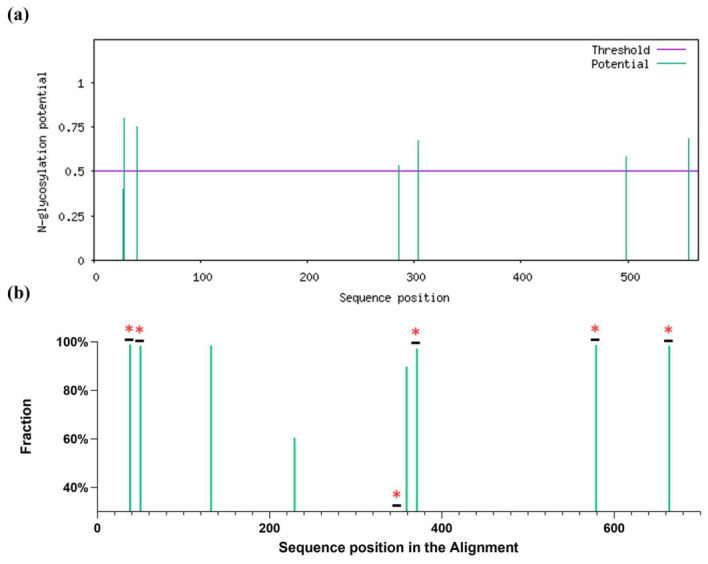
Prediction of glycosylation sites in the PR8 strain sequence (**a**); High-frequency glycosylation sites in the H1N1 strain (>60%, numbered according to PR8 after alignment) Glycosylation sites of PR8 marked with * (**b**).

Prediction of N-linked glycosylation sites in the PR8 HA sequence idetified six sites exceeding the software threshold (Figure 1a), representing a moderate glycosylation level relative to the overall population of influenza viruses. Comparative analysis of H1N1 sequences indicates that most strains harbor 6–8 glycosylation sites, with high-frequency positions summarized in Figure 1b (see Figure S3 for full analysis).

**Figure 2:**
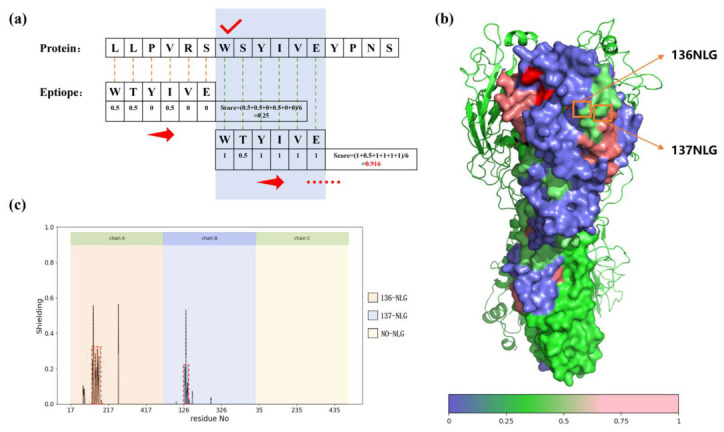
Schematic diagram of epitope and protein sequence alignment scoring (**a**); schematic diagram of epitope-enriched regions, where higher scores indicate higher frequency of amino acids in epitopes; and two enriched regions are highlighted (residues labeled in pink), including one proximal to the receptor-binding site (RBS; residues labeled in red) and one on the lateral surface (**b**); computational simulation of glycan shielding effects upon adding glycosylation at positions 136 and 137 (**c**).

B-cell epitopes of influenza viruses were retrieved from the IEDB and mapped onto the HA structure following the workflow shown in Figure 2a (Linear B-cell epitopes are treated as a sliding window of fixed length, traversed from the N-terminus of the HA protein in single-amino-acid steps. After each sliding step, the epitope is aligned residue-by-residue with the corresponding amino acid segment of the protein). Two epitope-enriched regions were identified within the HA1 head domain: one proximal to the receptor-binding site (RBS) and another located on the lateral surface (Figure 2b). Integration of epitope distribution with glycosylation site analysis yielded five candidate positions for glycan introduction. Among these, residues 136 and 137 were predicted to provide the most effective steric shielding of the lateral epitope-enriched region. (provides broader coverage and is closer to the lateral side epitope-enriched region). Glycoshield simulations further confirmed that glycosylation at these positions effectively masks the exposed epitopes (Figure 2c), whereas other candidate glycosylation sites showed comparatively limited shielding effects (Figure S4).

### 3.2. Glycosylation Enhances Protein Stability but Attenuates Viral Replication

The role of HA glycosylation in modulating acid and thermal stability has been well established [30,31]. Temperature, a key environmental and host factor, significantly influences IAV infectivity and tropism [32,33].

**Figure 3:**
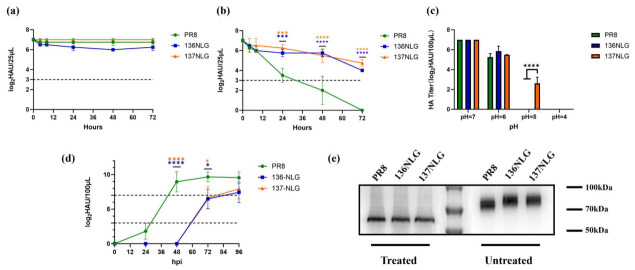
Temperature stability of PR8 and two glycosylation variants at 37 °C (**a**) and 42 °C (**b**), pH stability (**c**), viral growth kinetics at a low MOI (0.0001) (**d**), and identification of glycosylation modifications by Western blot (**e**). Lanes 1–3 and 5–7 show viral HA protein treated with- and withoutPNGase F, respectively. The markers in lane 4 (from top to bottom) correspond to 100 kDa, 70 kDa, and 50 kDa. *, *p* < 0.05; ***, *p* < 0.001; ****, *p* < 0.0001.

As shown in Figure 3a,b, all viruses maintained hemagglutination titers at 37 °C. However, after 72 h at 42 °C, the hemagglutination titer of wild-type PR8 fell below the detection limit, whereas both glycosylation mutants (136NLG and 137NLG) retained measurable activity, indicating enhanced thermostability. Under acidic conditions, hemagglutination titers decreased for all viruses relative to neutral pH; notably, the 137NLG mutant exhibited the greatest acid stability (Figure 3c). On the other hand, at a low MOI (0.0001), both mutants displayed delayed replication kinetics, with no detectable hemagglutination activity at 48 h post-infection (Figure 3d), suggesting that glycosylation impairs viral growth.

To confirm the glycosylation at the introduced NGSs, HA proteins were analyzed by Western blot following treatment with or without peptide-N-glycosidase F (PNGase F). In untreated samples, glycosylated HA proteins exhibited reduced electrophoretic mobility compared with the wild type, consistent with increased glycan occupancy. Following PNGase F treatment, all HA proteins migrated at similar positions (Figure 3e), confirming that the observed band shifts were attributable to NGSs at positions 136 and 137.

### 3.3. Glycosylation Modulates Viral Entry by Reducing HA-Sialic Acid Affinity

**Figure 4:**
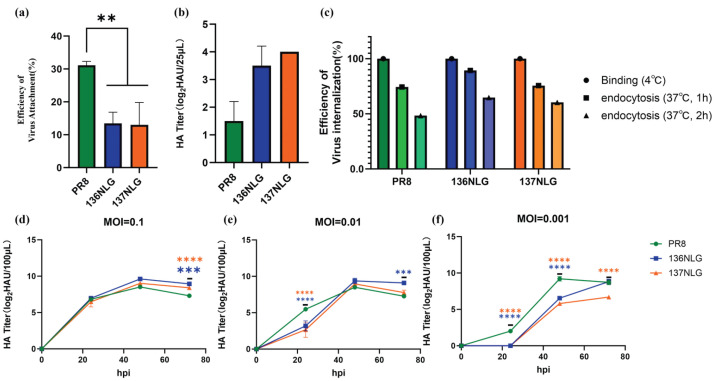
Virus adsorption efficiency (**a**) and virus adsorption-rerelease assays (**b**) implied reduced HA-Sialic acid affinity in the mutant strain. Virus internalization efficiency (**c**) and viral growth kinetics at different multiplicity of infection (MOI) (**d**–**f**).**, *p* < 0.01; ***, *p* < 0.001; ****, *p* < 0.0001.

To determine how HA glycosylation affects viral receptor binding, viral adsorption to MDCK cells and binding to chicken erythrocytes were evaluated using cell-based adsorption assays and adsorption–elution assays, respectively (Figure 4a,b). At equivalent MOI, both 136NLG and 137NLG mutants exhibited reduced adsorption to MDCK cells and more rapid elution from chicken erythrocytes compared with the wild-type virus, suggesting decreased HA–sialic acid binding affinity. Consistent with this observation, enhanced ELISA performed on virus-infected cells showed reduced internalization efficiency following adsorption in the mutant viruses (Figure 4c), indicating that impaired receptor binding contributes to delayed entry and subsequent replication kinetics [34].

To assess whether increased viral input could compensate for reduced receptor affinity, MDCK cells were infected at multiple MOIs and viral replication curves were determined (Figure 4d–f). The replication delay observed at low MOI progressively diminished with increasing MOI. At MOI = 0.1, no significant difference in hemagglutination titers was observed between mutants and the wild-type virus at 24 hpi, suggesting partial compensation by increased initial viral input.

These findings are consistent with viral kinetic principles: reduced receptor affinity lowers the adsorption rate constant, resulting in fewer initially infected cells at low MOI. At higher MOI, the increased probability of infection offsets this limitation, thereby minimizing differences in overall replication kinetics.

### 3.4. Glycosylation-Modified Vaccines Enhance Humoral Immunity in Mice

To evaluate the immunogenicity of glycosylation-modified viruses, BALB/c mice were immunized with inactivated whole-virus vaccines containing 0.135 μg HA per dose. Sera were collected at 14 days post-immunization for hemagglutination inhibition (HI) and microneutralization (MN) assays, followed by lethal challenge.

**Figure 5:**
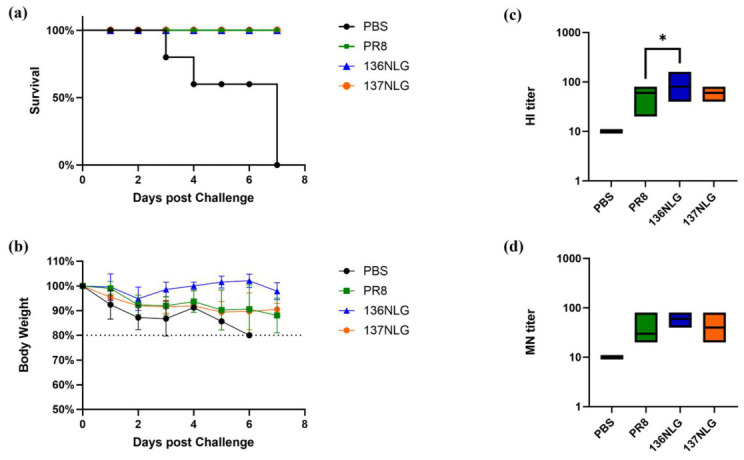
BALB/c mice were challenged intranasally with a lethal dose (10 × LD_50_) of PR8 influenza virus on day 14 post immunization. The survival rates (**a**) and body weight changes (**b**) of the mice (*n* = 3) were monitored for 7 days. The HI detection (**c**) and the MN antibody levels (**d**) (*n* = 6). Data significance was determined using one-way ANOVA.*, *p* < 0.05.

All vaccine groups conferred complete protection against mortality (Figure 5a). Notably, morbidity differed among groups: mice immunized with 136NLG exhibited reduced weight loss and faster recovery compared with the wild-type (WT) group (Figure 5b), indicating improved protective efficacy. Consistently, the 136NLG group induced a 1.78-fold higher geometric mean hemagglutination inhibition titer (GMT) than WT (89.8 vs. 50.4, *p* ＜ 0.05, 95% CI: 1.04–3.24), along with elevated MN antibody response (Figure 5c,d).The raw data supporting this figure are available in Supplementary Tables S4–S6.

In contrast, the 137NLG vaccine did not produce a measurable improvement in protection or weight loss relative to WT, although HI titers were modestly increased. This discrepancy may reflect differences in glycan-mediated epitope masking efficiency, as suggested by structural simulations (Figure 2c), but requires further validation. Overall, these results indicate that site-specific glycosylation at residue 136 enhances humoral immune responses and improves protection against disease severity, supporting its potential utility in influenza vaccine design.

## 4. Discussion

Our findings demonstrate that introduction of N-linked glycosylation sites at residues sites 136 and 137 of the HA protein in the PR8 strain is achievable through single-point mutations. These modifications enhance acid and thermal stability while reducing HA-sialic affinity, thereby impairing viral adsorption and internalization. When formulated as inactivated vaccines, the 136NLG variant conferred improved protection against disease severity, as evidenced by reduced weight loss in mice, compared with the wild-type strain.

Glycosylation has been widely explored in vaccine design for multiple viral pathogens, including influenza virus, HIV, SARS-CoV-2, and RSV [35,36,37,38,39,40,41]. In influenza, glycan engineering has been shown to modulate antigenicity and improve immunogenicity such as H7N9. For example, introduction of additional glycosylation sites in H7N9 HA enhanced vaccine efficacy, potentially by increasing HA incorporation into viral particles [42]. However, the relationship between glycosylation and immunogenicity is highly context-dependent, influenced by glycan number, position, and structural accessibility. Notably, partially glycosylated HA constructs have been reported to elicit stronger and more broadly neutralizing antibody responses than fully glycosylated counterparts, highlighting the importance of precise glycan placement [43].

Antigenic determinants of HA are predominantly located in the head domain, particularly in regions surrounding the receptor-binding site (RBS) [44,45,46,47]. While glycan addition near the RBS can sterically hinder receptor engagement, excessive interference may compromise viral fitness and propagation. Therefore, selective targeting of epitope-enriched regions distal to the RBS represents a rational strategy to balance immunogenicity and functionality [48]. In this study, integration of epitope mapping (IEDB) with structural analysis enabled the identification of candidate sites that maximize epitope shielding while preserving viral viability. This workflow provides a scalable framework for glycan-guided antigen design.

Mechanistically, the enhanced stability observed in glycosylated variants is consistent with the known role of glycans in stabilizing protein folding intermediates and reducing aggregation. However, glycosylation at residues 136 and 137, located within the HA head domain, also reduced receptor binding affinity, underscoring a trade-off between stability and infectivity. Quantitative characterization of binding affinity using approaches such as surface plasmon resonance will be required to further delineate these effects.

Interestingly, previous studies have shown that reducing or abolishing HA–receptor interactions can enhance humoral immune responses, potentially by improving antigen trafficking to lymphoid tissues and limiting non-specific binding [49]. Our findings are consistent with this concept, as reduced receptor affinity in the 136NLG variant was associated with enhanced antibody responses and improved protection against morbidity. In addition, the 136NLG variant may also enhance humoral immune responses by preserving or increasing the exposure of neutralizing epitopes, or by stabilizing favorable conformations of HA [50].

On the other hand, from a biophysical perspective, HA stability is a critical determinant of viral fitness and vaccine performance. Acid stability governs the pH threshold for membrane fusion, while thermal stability influences environmental persistence and vaccine storage. Enhancing HA stability may therefore improve both viral robustness and vaccine resilience, particularly under suboptimal cold-chain conditions [51,52,53].

Despite these promising results, several limitations should be noted. From the perspective of data and structural information, the proposed strategy relies on the availability of high-quality epitope datasets, which may limit its applicability to less-characterized or emerging viral strains. In addition, the current analysis primarily focuses on linear epitope distributions, whereas conformational epitopes—determined by three-dimensional protein structures—also play a critical role in immune recognition. Furthermore, the functional impact of glycosylation depends not only on site occupancy but also on glycan composition and structure; therefore, detailed characterization of site-specific glycans using techniques such as mass spectrometry and isotope labeling will be necessary to fully elucidate their effects on epitope masking and immunogenicity. Finally, studies have revealed that the major antigenic sites (Sa, Sb, Ca1, Ca2, Cb) on the HA surface exhibit a dynamically changing immunodominance hierarchy during infection (specifically characterized by early dominance of the Cb site and a gradual rise of the Sb and Sa sites at later stages) [54]. Future structural analyses will need to further integrate the limitations of current data regarding temporal dynamics, differences in immunization routes, and functional characterization of polyclonal sera, in order to more fully leverage the advantages of immunofocusing in enhancing vaccine efficacy. From an algorithmic perspective, the alignment-based epitope mapping approach could be further refined. Incorporation of structural constraints and advanced computational methods, such as machine learning, may improve predictive accuracy. For example, residues with known functional importance—such as those involved in MHC binding or located at structurally conserved regions—could be more rationally weighted during alignment. Integrating sequence, structural, and functional information into a unified framework would likely enhance the robustness and generalizability of this strategy.

Notably, the immunization regimen in this study differs from the conventional schedule, and the sample size in the challenge experiment was relatively small (*n* = 3). Although serological data demonstrated statistically significant differences among the groups, subsequent studies will still require increasing the number of animals to enhance the reliability of the challenge experiment, conducting heterologous challenge experiments and evaluating serum cross-reactivity to analyze the impact of glycosylation modifications on the breadth of vaccine protection, extending the duration of body weight monitoring, and adopting a prime-boost regimen to further investigate the persistence of protective efficacy.

In conclusion, this study provides mechanistic insights into the role of glycosylation in modulating HA stability, receptor binding, and immunogenicity. The proposed structure-guide glycan design strategy offers a rational framework for improving influenza vaccine efficacy and may be broadly applicable to the development of next-generation vaccines.

## Data Availability

The data presented in this study are available in this article and the Supplementary Materials.

## Supplementary Materials

The following supporting information can be downloaded at: https://www.mdpi.com/article/10.3390/vaccines14050443/s1. The following supporting information can be downloaded for free. (Table S1: Positive B-cell epitope of influenza download from IEDB; Table S2: The scores for all amino acids following alignment; Table S3: Statistical analysis of normalized scores following alignment of epitopes with the HA protein; Table S4: Raw data of HI; Table S5: Raw data of MN.; Table S6: Raw data of challenge experiment. Figure S1: Eight plasmids for the WT H1N1 (A/PR8/34) strain of IAV; Figure S2: Point mutations and the introduction of glycosylation motifs; Figure S3: Analysis of glycosylation sites in H1N1 influenza; Figure S4: Predicted glycosylation coverage of potential glycosylation sites in Glycoshield, using the high-mannose glycoform as a simulated grafted glycan chain; Figure S5: Computational simulation of glycan shielding effects upon adding glycosylation at positions 142, 143 and 144); Figure S6: Original WB image, untreated with PNGase-F; Figure S7: Original WB image, treated with PNGase-F..

## Abbreviations

The following abbreviations are used in this manuscript:

HA	Hemagglutinin
NA	Neuraminidase
NP	Nucleoprotein
NS	Non-structural Protein
PB1	Polymerase Basic Protein 1
PB2	Polymerase Basic Protein 2
M	Matrix Protein
IEDB	Immune Epitope Database
IAV	Influenza A virus
HI	Hemagglutination Inhibition Assay
MN	Microneutralization Assay
WT	Wild Type
MOI	Multiplicity of Infection
PR8	A/Puerto Rico/8/1934(H1N1)
RDE	Receptor Destroying Enzyme
DMEM	Dulbecco’s Modified Eagle’s Medium
NLG	N-linked glycosylation
NGS	N-linked glycosylation sites

## References

[1] Lycett S.J., Duchatel F., Digard P. (2019). A Brief History of Bird Flu. Philos. Trans. R. Soc. Lond. B Biol. Sci..

[2] Krammer F., Smith G.J.D., Fouchier R.A.M., Peiris M., Kedzierska K., Doherty P.C., Palese P., Shaw M.L., Treanor J., Webster R.G. (2018). Influenza. Nat. Rev. Dis. Prim..

[3] Boivin W., Loeb M., Openshaw P., Ashraf M., Pawelec G. (2025). Seasonal Influenza Vaccination: Overcoming Immunosenescence with Enhanced Vaccines. Vaccine X.

[4] Musunuri S., Weidenbacher P.A.B., Kim P.S. (2024). Bringing Immunofocusing into Focus. npj Vaccines.

[5] Harris A., Cardone G., Winkler D.C., Heymann J.B., Brecher M., White J.M., Steven A.C. (2006). Influenza Virus Pleiomorphy Characterized by Cryoelectron Tomography. Proc. Natl. Acad. Sci. USA.

[6] Kornfeld R., Kornfeld S. (1985). Assembly of Asparagine-Linked Oligosaccharides. Annu. Rev. Biochem..

[7] Kim J.I., Park M.-S. (2012). N-Linked Glycosylation in the Hemagglutinin of Influenza A Viruses. Yonsei Med. J..

[8] Sun X., Jayaraman A., Maniprasad P., Raman R., Houser K.V., Pappas C., Zeng H., Sasisekharan R., Katz J.M., Tumpey T.M. (2013). N-Linked Glycosylation of the Hemagglutinin Protein Influences Virulence and Antigenicity of the 1918 Pandemic and Seasonal H1N1 Influenza A Viruses. J. Virol..

[9] Dosey A., Ellis D., Boyoglu-Barnum S., Syeda H., Saunders M., Watson M.J., Kraft J.C., Pham M.N., Guttman M., Lee K.K. (2023). Combinatorial Immune Refocusing within the Influenza Hemagglutinin RBD Improves Cross-Neutralizing Antibody Responses. Cell Rep..

[10] Bajic G., Maron M.J., Adachi Y., Onodera T., McCarthy K.R., McGee C.E., Sempowski G.D., Takahashi Y., Kelsoe G., Kuraoka M. (2019). Influenza Antigen Engineering Focuses Immune Responses to a Subdominant but Broadly Protective Viral Epitope. Cell Host Microbe.

[11] Hoffmann E., Neumann G., Kawaoka Y., Hobom G., Webster R.G. (2000). A DNA Transfection System for Generation of Influenza A Virus from Eight Plasmids. Proc. Natl. Acad. Sci. USA.

[12] Kalbfuss B., Knöchlein A., Kröber T., Reichl U. (2008). Monitoring Influenza Virus Content in Vaccine Production: Precise Assays for the Quantitation of Hemagglutination and Neuraminidase Activity. Biologicals.

[13] Tzeng T.-T., Chen P.-L., Weng T.-C., Tsai S.-Y., Lai C.-C., Chou H.-I., Chen P.-W., Lu C.-C., Liu M.-T., Sung W.-C. (2020). Development of High-Growth Influenza H7N9 Prepandemic Candidate Vaccine Viruses in Suspension MDCK Cells. J. Biomed. Sci..

[14] Karakus U., Crameri M., Lanz C., Yángüez E. (2018). Propagation and Titration of Influenza Viruses. Methods Mol. Biol..

[15] Wang Q., Luo J., Li B., Ye Q., Xu W., Gao F., Zhou L., Lu W., Tan W.-S., Li X. (2024). Reduction in Interferon-Stimulated Genes Contributes to High-Yield Production of Influenza Virus in Suspension MDCK Cells. Vaccines.

[16] Zhou Z.-J., Qiu Y., Pu Y., Huang X., Ge X.-Y. (2020). BioAider: An Efficient Tool for Viral Genome Analysis and Its Application in Tracing SARS-CoV-2 Transmission. Sustain. Cities Soc..

[17] Katoh K., Misawa K., Kuma K., Miyata T. (2002). MAFFT: A Novel Method for Rapid Multiple Sequence Alignment Based on Fast Fourier Transform. Nucleic Acids Res..

[18] Zhang M., Gaschen B., Blay W., Foley B., Haigwood N., Kuiken C., Korber B. (2004). Tracking Global Patterns of N-Linked Glycosylation Site Variation in Highly Variable Viral Glycoproteins: HIV, SIV, and HCV Envelopes and Influenza Hemagglutinin. Glycobiology.

[19] Gupta R., Brunak S. (2001). Prediction of Glycosylation across the Human Proteome and the Correlation to Protein Function. Biocomputing 2002.

[20] Blazeska N., Kosaloglu-Yalcin Z., Vita R., Peters B., Sette A. (2023). IEDB and CEDAR: Two Sibling Databases to Serve the Global Scientific Community. Methods Mol. Biol..

[21] Tsai Y.-X., Chang N.-E., Reuter K., Chang H.-T., Yang T.-J., von Bülow S., Sehrawat V., Zerrouki N., Tuffery M., Gecht M. (2024). Rapid Simulation of Glycoprotein Structures by Grafting and Steric Exclusion of Glycan Conformer Libraries. Cell.

[22] Milder F.J., Jongeneelen M., Ritschel T., Bouchier P., Bisschop I.J.M., de Man M., Veldman D., Le L., Kaufmann B., Bakkers M.J.G. (2022). Universal Stabilization of the Influenza Hemagglutinin by Structure-Based Redesign of the pH Switch Regions. Proc. Natl. Acad. Sci. USA.

[23] Wen F., Li L., Zhao N., Chiang M.-J., Xie H., Cooley J., Webby R., Wang P.G., Wan X.-F. (2018). A Y161F Hemagglutinin Substitution Increases Thermostability and Improves Yields of 2009 H1N1 Influenza A Virus in Cells. J. Virol..

[24] Sun Y., Zhu Y., Zhang P., Sheng S., Guan Z., Cong Y. (2024). Hemagglutinin Glycosylation Pattern-Specific Effects: Implications for the Fitness of H9.4.2.5-Branched H9N2 Avian Influenza Viruses. Emerg. Microbes Infect..

[25] Qian Y., Hong Y., Zhi X., Liang Z., Tan W.-S. (2025). Insights into IAV Replication and Lipid Metabolism in Suspension-Adapted MDCK-STAT1-KO Cells. Vaccines.

[26] Hirst G.K. (1942). Adsorption of Influenza Hemagglutinins and Virus by Red Blood Cells. J. Exp. Med..

[27] Glauser D.L., Milho R., Frederico B., May J.S., Kratz A.-S., Gillet L., Stevenson P.G. (2013). Glycoprotein B Cleavage Is Important for Murid Herpesvirus 4 To Infect Myeloid Cells. J. Virol..

[28] Zhu H., Lee A.C.Y., Li C., Mak W.W.N., Chen Y.Y., Chan K.-H., Zhang A.J.X., Fung W.-F., Zhang R.-Q., Fung Y.-F. (2018). Low Population Serum Microneutralization Antibody Titer against the Predominating Influenza A(H3N2) N121K Virus during the Severe Influenza Summer Peak of Hong Kong in 2017. Emerg. Microbes Infect..

[29] Zhu R., Xu S., Sun W., Li Q., Wang S., Shi H., Liu X. (2022). HA Gene Amino Acid Mutations Contribute to Antigenic Variation and Immune Escape of H9N2 Influenza Virus. Vet. Res..

[30] Yin Y., Zhang X., Qiao Y., Wang X., Su Y., Chen S., Qin T., Peng D., Liu X. (2017). Glycosylation at 11Asn on Hemagglutinin of H5N1 Influenza Virus Contributes to Its Biological Characteristics. Vet. Res..

[31] Sun H., Deng G., Sun H., Song J., Zhang W., Li H., Wei X., Li F., Zhang X., Liu J. (2022). N-Linked Glycosylation Enhances Hemagglutinin Stability in Avian H5N6 Influenza Virus to Promote Adaptation in Mammals. PNAS Nexus.

[32] Kaverin N.V., Rudneva I.A., Smirnov Y.A., Finskaya N.N. (1988). Human-Avian Influenza Virus Reassortants: Effect of Reassortment Pattern on Multi-Cycle Reproduction in MDCK Cells. Arch. Virol..

[33] Scull M.A., Gillim-Ross L., Santos C., Roberts K.L., Bordonali E., Subbarao K., Barclay W.S., Pickles R.J. (2009). Avian Influenza Virus Glycoproteins Restrict Virus Replication and Spread through Human Airway Epithelium at Temperatures of the Proximal Airways. PLoS Pathog..

[34] Wu N.C., Wilson I.A. (2020). Influenza Hemagglutinin Structures and Antibody Recognition. Cold Spring Harb. Perspect. Med..

[35] Swanson K.A., Rainho-Tomko J.N., Williams Z.P., Lanza L., Peredelchuk M., Kishko M., Pavot V., Alamares-Sapuay J., Adhikarla H., Gupta S. (2020). A Respiratory Syncytial Virus (RSV) F Protein Nanoparticle Vaccine Focuses Antibody Responses to a Conserved Neutralization Domain. Sci. Immunol..

[36] Ringe R.P., Pugach P., Cottrell C.A., LaBranche C.C., Seabright G.E., Ketas T.J., Ozorowski G., Kumar S., Schorcht A., van Gils M.J. (2019). Closing and Opening Holes in the Glycan Shield of HIV-1 Envelope Glycoprotein SOSIP Trimers Can Redirect the Neutralizing Antibody Response to the Newly Unmasked Epitopes. J. Virol..

[37] Ingale J., Tran K., Kong L., Dey B., McKee K., Schief W., Kwong P.D., Mascola J.R., Wyatt R.T. (2014). Hyperglycosylated Stable Core Immunogens Designed to Present the CD4 Binding Site Are Preferentially Recognized by Broadly Neutralizing Antibodies. J. Virol..

[38] Pantophlet R., Wilson I.A., Burton D.R. (2004). Improved Design of an Antigen with Enhanced Specificity for the Broadly HIV-Neutralizing Antibody B12. Protein Eng. Des. Sel..

[39] Li Y., Cleveland B., Klots I., Travis B., Richardson B.A., Anderson D., Montefiori D., Polacino P., Hu S.-L. (2008). Removal of a Single N-Linked Glycan in Human Immunodeficiency Virus Type 1 Gp120 Results in an Enhanced Ability to Induce Neutralizing Antibody Responses. J. Virol..

[40] Eggink D., Goff P.H., Palese P. (2014). Guiding the Immune Response against Influenza Virus Hemagglutinin toward the Conserved Stalk Domain by Hyperglycosylation of the Globular Head Domain. J. Virol..

[41] Lin S.-C., Lin Y.-F., Chong P., Wu S.-C. (2012). Broader Neutralizing Antibodies against H5N1 Viruses Using Prime-Boost Immunization of Hyperglycosylated Hemagglutinin DNA and Virus-Like Particles. PLoS ONE.

[42] Kim J.I., Park S., Bae J.-Y., Lee S., Kim J., Kim G., Yoo K., Heo J., Kim Y.S., Shin J.S. (2020). Glycosylation Generates an Efficacious and Immunogenic Vaccine against H7N9 Influenza Virus. PLoS Biol..

[43] Tseng Y.-C., Wu C.-Y., Liu M.-L., Chen T.-H., Chiang W.-L., Yu Y.-H., Jan J.-T., Lin K.-I., Wong C.-H., Ma C. (2019). Egg-Based Influenza Split Virus Vaccine with Monoglycosylation Induces Cross-Strain Protection against Influenza Virus Infections. Proc. Natl. Acad. Sci. USA.

[44] Lewis N.S., Daly J.M., Russell C.A., Horton D.L., Skepner E., Bryant N.A., Burke D.F., Rash A.S., Wood J.L.N., Chambers T.M. (2011). Antigenic and Genetic Evolution of Equine Influenza A (H3N8) Virus from 1968 to 2007. J. Virol..

[45] Lewis N.S., Russell C.A., Langat P., Anderson T.K., Berger K., Bielejec F., Burke D.F., Dudas G., Fonville J.M., Fouchier R.A. (2016). The Global Antigenic Diversity of Swine Influenza A Viruses. eLife.

[46] Hensley S.E., Das S.R., Bailey A.L., Schmidt L.M., Hickman H.D., Jayaraman A., Viswanathan K., Raman R., Sasisekharan R., Bennink J.R. (2009). Hemagglutinin Receptor Binding Avidity Drives Influenza A Virus Antigenic Drift. Science.

[47] Li J., Liu S., Gao Y., Tian S., Yang Y., Ma N. (2021). Comparison of N-Linked Glycosylation on Hemagglutinins Derived from Chicken Embryos and MDCK Cells: A Case of the Production of a Trivalent Seasonal Influenza Vaccine. Appl. Microbiol. Biotechnol..

[48] Hendin H.E., Lavoie P.-O., Gravett J.M., Pillet S., Saxena P., Landry N., D’Aoust M.-A., Ward B.J. (2022). Elimination of Receptor Binding by Influenza Hemagglutinin Improves Vaccine-Induced Immunity. npj Vaccines.

[49] Liu D.J., Zhong X.Q., Ru Y.X., Zhao S.L., Liu C.C., Tang Y.B., Wu X., Zhang Y.S., Zhang H.H., She J.Y. (2024). Disulfide-stabilized trimeric hemagglutinin ectodomains provide enhanced heterologous influenza protection. Emerg. Microbes Infect..

[50] Carr C.M., Chaudhry C., Kim P.S. (1997). Influenza Hemagglutinin Is Spring-Loaded by a Metastable Native Conformation. Proc. Natl. Acad. Sci. USA.

[51] Russell C.J., Hu M., Okda F.A. (2018). Influenza Hemagglutinin Protein Stability, Activation, and Pandemic Risk. Trends Microbiol..

[52] Russier M., Yang G., Briard B., Meliopoulos V., Cherry S., Kanneganti T.-D., Schultz-Cherry S., Vogel P., Russell C.J. (2020). Hemagglutinin Stability Regulates H1N1 Influenza Virus Replication and Pathogenicity in Mice by Modulating Type I Interferon Responses in Dendritic Cells. J. Virol..

[53] Leung V., Mapletoft J., Zhang A., Lee A., Vahedi F., Chew M., Szewczyk A., Jahanshahi-Anbuhi S., Ang J., Cowbrough B. (2019). Thermal Stabilization of Viral Vaccines in Low-Cost Sugar Films. Sci. Rep..

[54] Angeletti D., Gibbs J., Angel M., Kosik I., Hickman H.D., Frank G.M., Das S.R., Wheatley A.K., Prabhakaran M., Leggat D.J. (2017). Defining B cell immunodominance to viruses. Nat. Immunol..

