# Supplementary material for: Structure-Guided Glycosylation of Hemagglutinin Enhances Stability and Modulates Immunogenicity of Influenza Vaccines
Source: Vaccines (Basel). 2026 May 15;14(5):443. doi: 10.3390/vaccines14050443 (PMC13211694; doi:10.3390/vaccines14050443)
Supplement: Supplementary file 1 [file vaccines-14-00443-s001.zip › vaccines-4240461-supplementary.pdf]

Supplementary Materials

Name	Coding for
CH-PB2	vRNA and mRNA of PB2
CH-PB1	vRNA and mRNA of PB1
CH-PA	vRNA and mRNA of PA
CH-NP	vRNA and mRNA of NP
CH-M	vRNA and mRNA of M
CH-NS	vRNA and mRNA of NS
CH-HA	vRNA and mRNA of HA
CH-NA	vRNA and mRNA of NA

Illustration

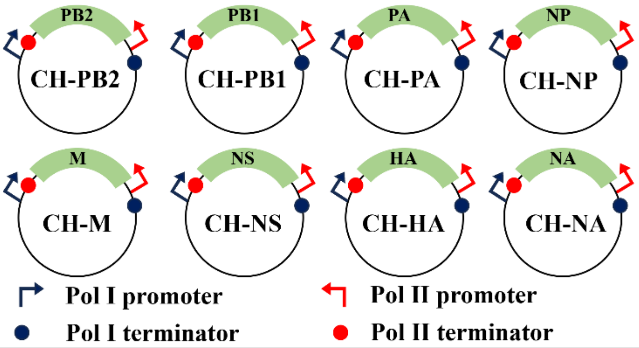

Figure S1. Eight plasmids (CH-PB2, CH-PB1, CH-PA, CH-M, CH-HA, CH-NA, CH-NP, and CH-NS) for the wt H1N1 (A/PR8/34) strain of IAV.

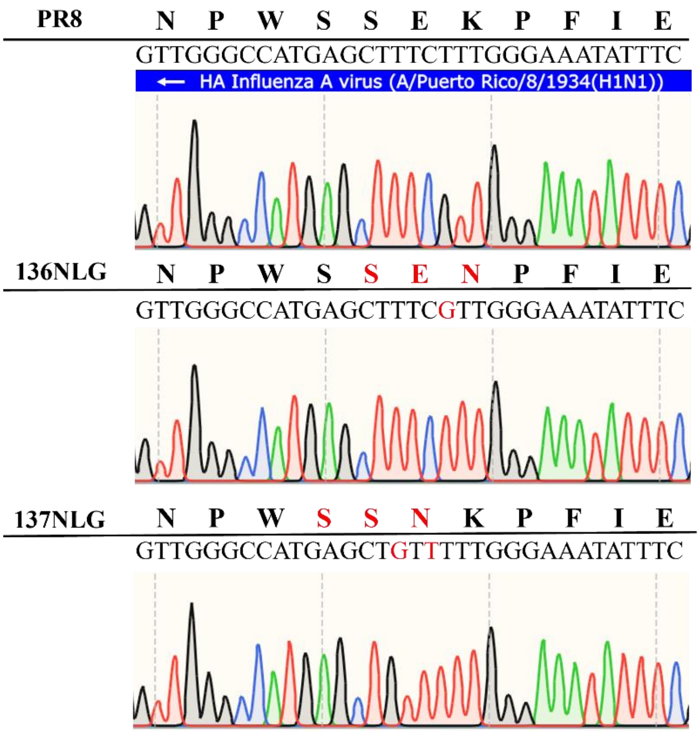

Figure S2. Point mutations and the introduction of glycosylation motifs, marked in red.

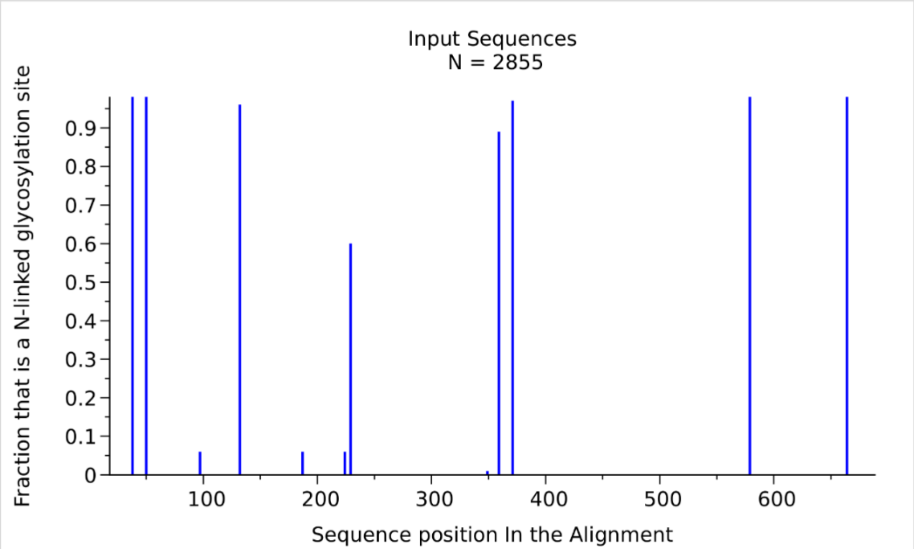

Figure S3. Analysis of glycosylation sites in H1N1 influenza.

Potential glycosylation sites	Predicted glycosylation coverage
136	46.38 %
137	75.38 %
142	16.33 %
143	0.33 %
144	8.73 %

Figure S4. Predicted glycosylation coverage of potential glycosylation sites in Glyshield, using the high-mannose glycoform as a simulated grafted glycan chain.

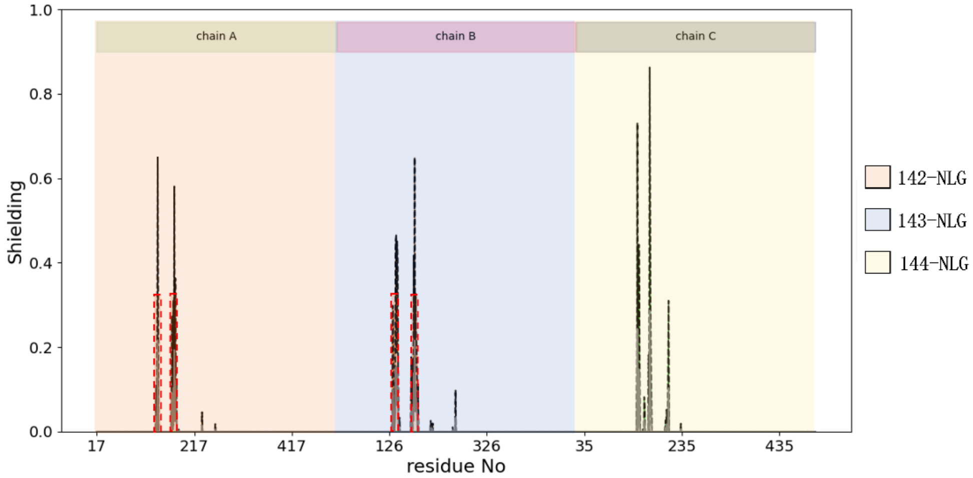

Figure S5. Computational simulation of glycan shielding effects upon adding glycosylation at positions 142,143 and 144.

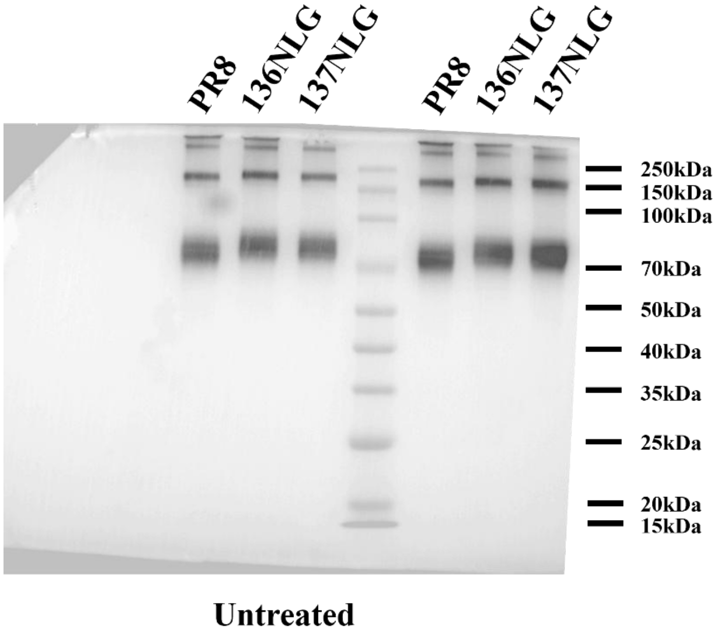

Figure S6. Original WB image, untreated with PNGase-F.

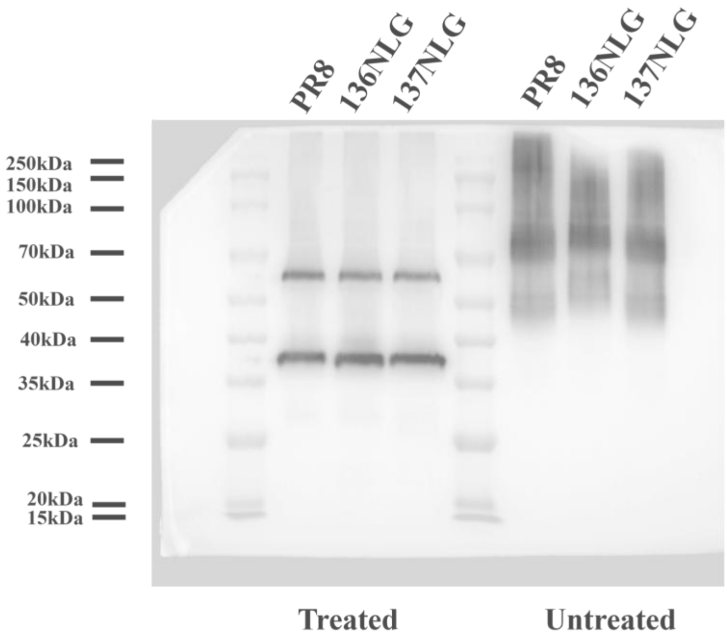

Figure S7. Original WB image, treated with PNGase-F.
